# Supplementary figures and images for: RORγt inverse agonists demonstrating a margin between inhibition of IL-17A and thymocyte apoptosis
Source: PLoS One. 2025 Jan 16;20(1):e0317090. doi: 10.1371/journal.pone.0317090 (PMC11737796; doi:10.1371/journal.pone.0317090)

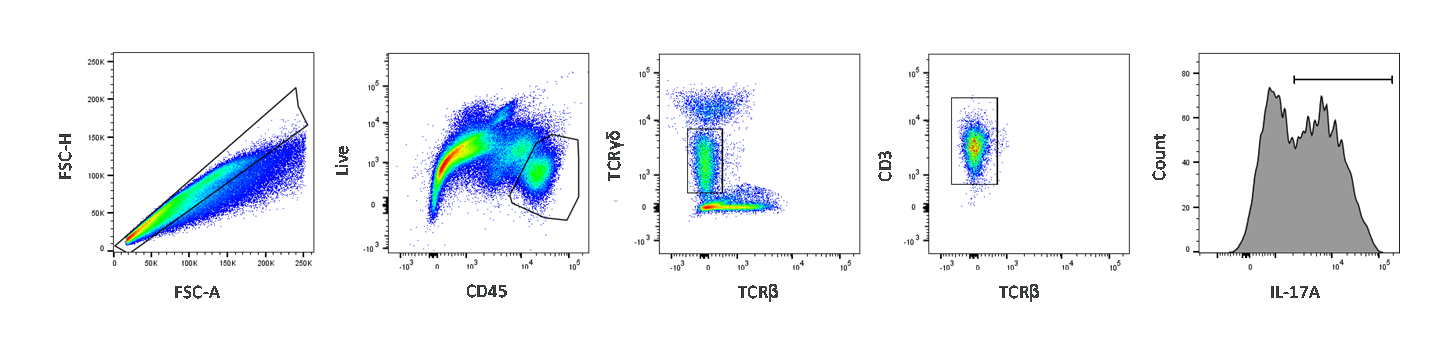

Supplement: S1 Fig — Single cells were selected followed by CD45+ live cells, TCRγδ intermediate TCRβ negative cells, further selected on CD3 and IL-17A using a histogram. (TIF) [file pone.0317090.s001.tif]

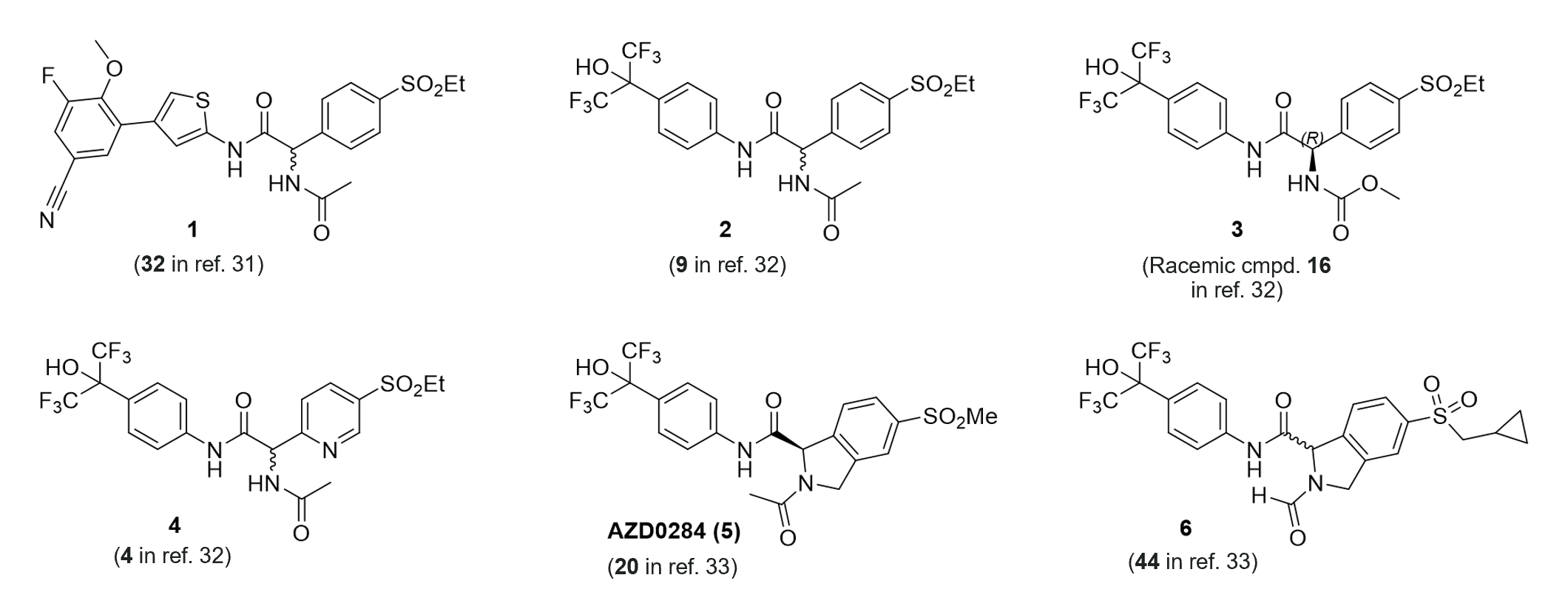

Supplement: S2 Fig — (TIF) [file pone.0317090.s002.tif]

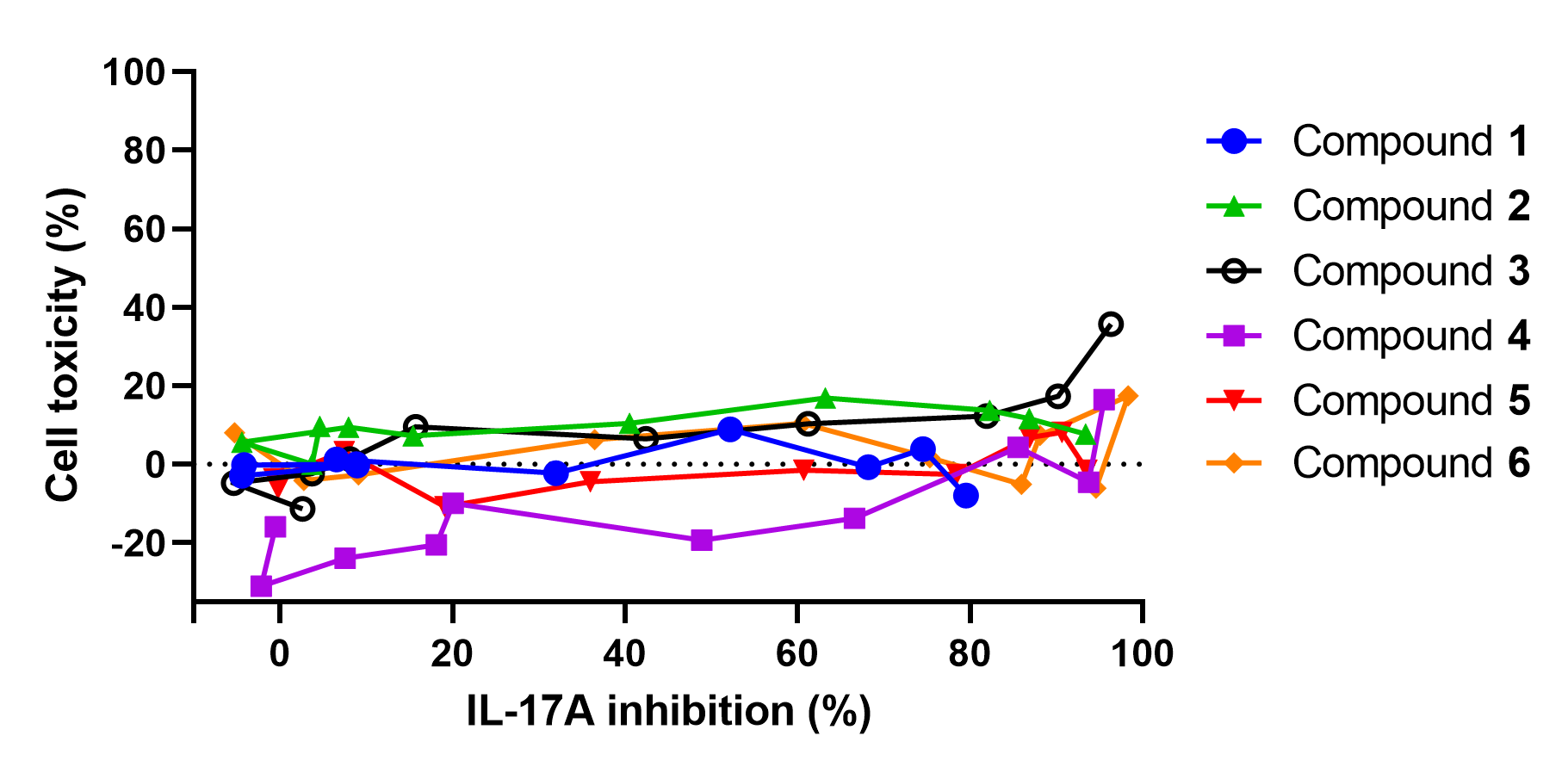

Supplement: S3 Fig — (TIF) [file pone.0317090.s003.tif]

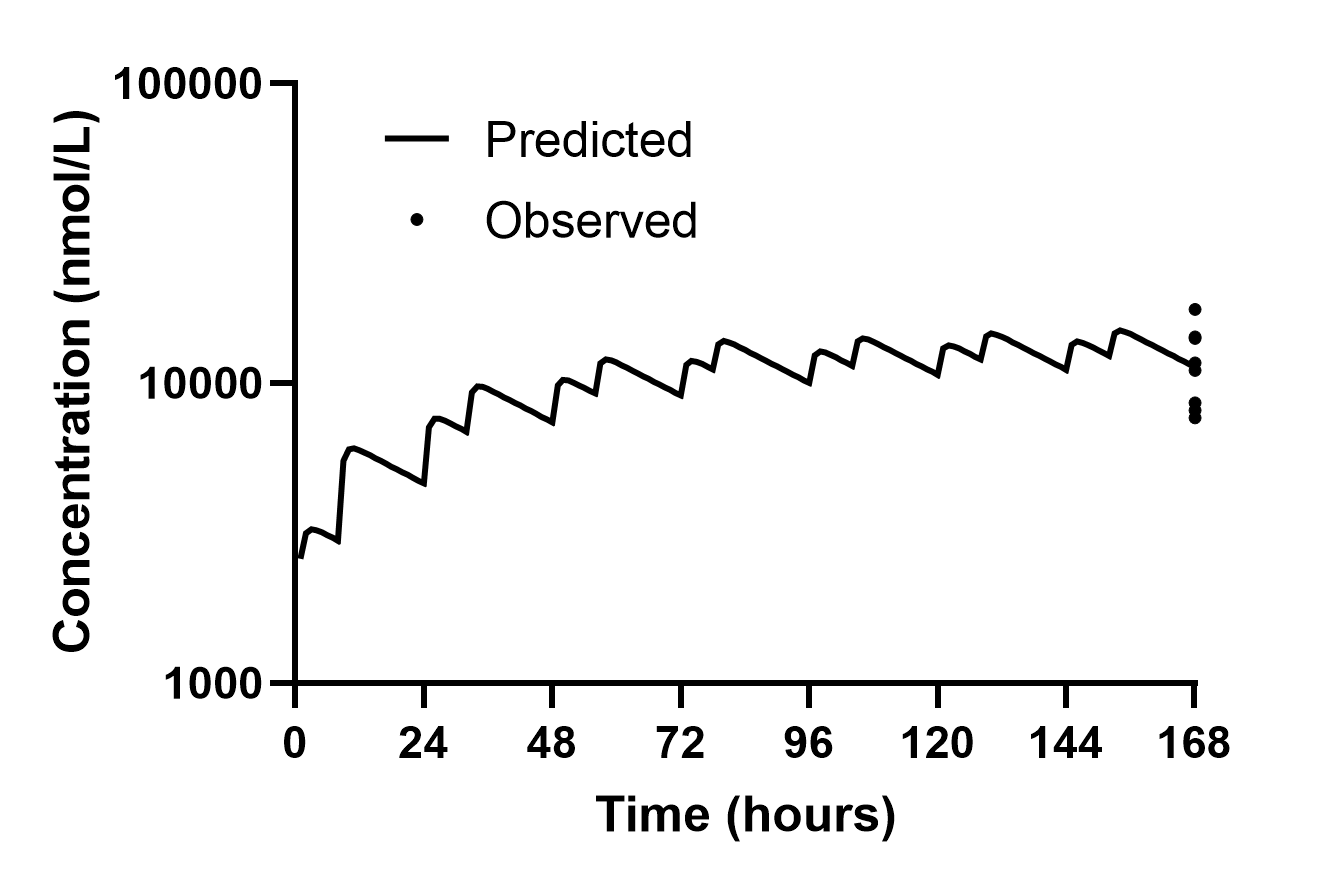

Supplement: S4 Fig — Exposure prediction (line) and terminal concentrations (dots) in eight mice treated with compound 3 following 7 days twice-daily oral dosing with 50 mg/kg in the IMQ-induced skin inflammation model. (TIF) [file pone.0317090.s004.tif]

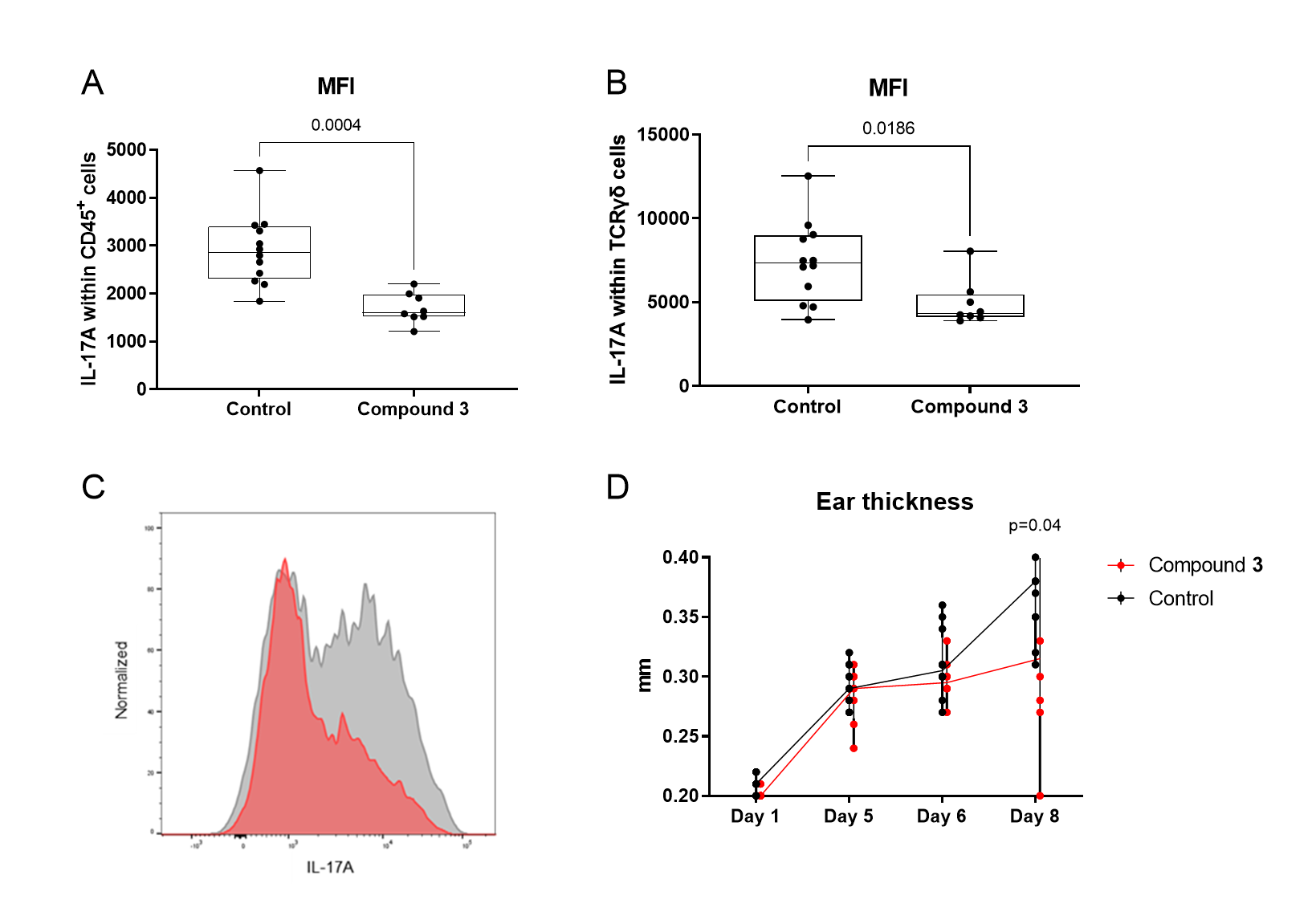

Supplement: S5 Fig — A) Mean fluorescence intensity of IL-17A in CD45+ cells B) Mean fluorescence intensity of IL-17A in γδ T cells C) Histogram of IL-17A in γδ T cells, grey: Vehicle, in red: Compound 3 D) Ear thickness over time with individual data points visualised. (TIF) [file pone.0317090.s005.tif]
