# Supplementary material for: RORγt inverse agonists demonstrating a margin between inhibition of IL-17A and thymocyte apoptosis
Source: PLoS One. 2025 Jan 16;20(1):e0317090. doi: 10.1371/journal.pone.0317090 (PMC11737796; doi:10.1371/journal.pone.0317090)
Supplement: Attachment — Submitted filename: Response to reviewers.docx [file pone.0317090.s007.docx]

ANSWERS TO REVIEWER’S COMMENTS

PONE-D-24-01191

**RORγt inverse agonists demonstrating a margin between inhibition of IL-17A and thymocyte apoptosis**

***Editor’s summary and main concerns:***

Thank you for submitting your manuscript to PLOS ONE. After careful consideration, we feel that it has merit but does not fully meet PLOS ONE’s publication criteria as it currently stands. Therefore, we invite you to submit a revised version of the manuscript that addresses the points raised during the review process.

Dear Editor

Thank you for the possibility to revise this manuscript. It is clear to us that the reviewers have spent substantial time and effort in reviewing our work in order to help us make improvements.

We have formulated responses to each of the questions below and updated the manuscript accordingly.

We believe that the paper is improved from the feedback we have received and that the text is clearer and easier to read and understand.

Yours sincerely,

Dr Mia Collins

Review Comments to the Author

**Reviewer 1:**

Reviewer #1: The manuscript "RORγt inverse agonists demonstrating a margin between inhibition of IL-17A and thymocyte apoptosis" investigates a fundamental duality in T cell functions; where targeting the key transcription factor that promotes Th17 cell functions in autoimmune diseases, may also lead to a defect in thymic T cell development. The work looks into the problem from a clinical perspective and finds a difference in dose for RORγt inverse agonists to exert these two opposite effects, which might be of therapeutic significance. Overall, the work has merit, but also has certain technical pitfalls. I have a few points for suggestions, as listed below.

a. The primary aim of this work is to uncouple CD4+CD8+ thymocyte apoptosis from Th17 cell activity modulation by the RORγt-targeted compounds. Since RORγt expression is thymus-specific, while RORγ is not expressed in the thymus to a considerable extent, the authors might have chosen a compound that selectively targets RORγ and not RORγt. This could have completely abolished any thymus-specific effect. The authors need to justify this.

Thank you for this comment, it is indeed a great suggestion and it would have been optimal to include such a compound. However, except for the compound referenced in the discussion of the manuscript (Reference 54 Polasek et al), we are not aware of any compound that distinguishes between the 2 isoforms and the referenced compound was not available at the time of the study. The protein sequence of the ligand binding domain is identical between the RORγ and RORγt isoforms, and isoform specificity is likely to be very hard to achieve. Furthermore, since both isoforms are expressed in Th17 cells, it is likely that it will be necessary to inhibit both RORγ and RORγt to achieve efficacy in peripheral T-cells. This data is stated in the introduction, rows 77-84, and we have added an additional clarification to accommodate the reviewers feedback rows 667-669.

b. IL-17 and other proinflammatory cytokine signalling pathways are highly active in early T cell progenitors and are associated with their leukemic potential (reference doi: 10.3389/fcell.2022.899752). Even though the authors have mentioned the implications of RORγt inhibition on thymic lymphoma in mice, they have not mentioned the potential effects of such therapies on the development of thymic T cell leukemia, which should be separately addressed as a limitation of the study.

This is an important point, and we share these thoughts and concerns. We have now made it even more clear than before that this might be a problem in humans and we added to this discussion at rows 646-649. The following section now reads:

*“Hence, today, clinical progression of this class of inhibitor seems to be an unsurmountable hurdle due to the lack of means to predict and evaluate the safety profile with regards to development of thymic lymphoma and T-ALL, which is a clear limitation.”*

Similarly, the authors have also not mentioned the importance of RORγt on the selection of diverse TCRs in the thymus, which is a major hindrance to autoimmunity (reference doi: 10.1016/j.celrep.2016.11.073). The potential risks of developing T cells with autoimmune features by RORγt inhibition should also be discussed.

We would like to thank the reviewer for this important point. We have updated the manuscript to reflect this data and its potential consequences. Rows 638-644 which now reads:

*“An additional complication of inhibition of RORgt has been suggested by Guo et al [51] where they have demonstrated a skewing of the TCRa gene rearrangement and hence limitations to the diversity of the T cell repertoire in mice. While skewing of the T cell repertoire is noteworthy, due to the potentially enhanced risk of developing autoimmune disorders and infection, the T cell leukopenia observed in humans carrying RORC biallelic loss of function mutations, is less pronounced than that observed in mice [19].”*

c. The ex vivo mouse thymocyte apoptosis assay in presence or absence of the compounds of interest has been performed without stimulating the thymocytes. However, apoptosis of thymocytes in the thymus during the selection checkpoints is dependent on a T cell receptor-mediated activation signal. The authors need to point out this fact and mention the need for assessing thymocyte apoptosis by these compounds upon ex vivo stimulation.

Thank you for this comment, it is important to bring up several aspects of this complicated biology. Thymocytes apoptose naturally in the thymus both due to an overly strong TCR engagement and a too week, or lack of engagement aka “death by neglect”. We have updated the text to reflect this and to make it more clear. Rows 67-69 now reads:

*“In the mouse, thymocytes deficient in RORγt remain hyperproliferative but they also rapidly apoptose through the lack of RORγt-dependent Bcl-xL induced quiescence [16, 18].”*

d. Even though the authors show significant success of compound 3 in reducing the levels of IL-17A as well as γδ T cells in the ear after imiquimod-induced skin inflammation, they might have also shown their levels in the systemic circulation (for example, in blood or spleen), to establish its role in moderating the systemic inflammation.

This is a great suggestion and in hindsight we might have included this. The IMQ model mainly induces a local inflammation in the ear during the 8-day treatment period. The point we would like to make with this experiment is to demonstrate that through peroral drug administration we achieve systemic exposure at sufficient levels to block inflammation even in the periphery such as the ear pinnas. As we have demonstrated exposure in the blood, we have no doubt that we will also affect inflammation throughout the body.

e. The authors have stated their inability to test their compound's efficacy in mediating thymocyte apoptosis during imiquimod-mediated skin inflammation. This could have been overcome by changing the dose of imiquimod which would have induced skin inflammation but with minimal damage to the thymus.

Alternatively, IL-17A-dependent established inflammatory models which do not cause significant damage to the thymus (reference doi: 10.1111/j.1365-3083.2007.01923.x), might have been used. The authors need to discuss this issue.

Thank you for these comments. While several processes affect thymocyte survival, we have not been able to find any data indicating that IMQ causes thymic damage. Ageing, however, comes with the involution of the thymus where the organ reduces in size with age, as well as its output of naïve T-cells. This process is variable in individuals and across the sexes. This variability hampers the possibility to use the thymic readouts in such models where ethical considerations dictate the usable age span of the experimental animals. Thymus involution sets in approximately at sexual maturation in mice-(6-8 weeks). Additionally, the daily application of IMQ creme and per oral compound administration induces some stress to the animals that affects the cellularity and weight of the animals’ thymae. In line with this reasoning, the suggested use of the dextran sulphate sodium-induced intestinal inflammation model is unlikely to aid in circumventing the stress, malaise and age dependent variability of the thymus size and cellularity. We have updated the section that highlights that this separation between thymic effects and effect on IMQ induced inflammation is important and subject to stress and age dependent variability. Rows 675-685 now read:

*“A further limitation is the lack of establishing an in vivo margin between the positive effect of RORγt inhibition (reduction of IL-17A) and the unwanted effect of thymocyte apoptosis in the same animal experiment. This was not achievable due to the stress-sensitive nature of the thymus which undergoes rapid involution upon handling (e.g application of IMQ cream and dosing of compounds) of the animals. Furthermore, the early onset of natural thymic involution in mice around sexual maturity (6-8 weeks) would introduce further variability in thymus size in the adult animals needed in the IMQ experiment, both for the immune system to be fully matured and to minimize variability from growth related differences in size and thickness of ear pinnas and cell numbers. This limitation did not apply to the experiments studying thymus effects as the animals were intentionally young to ensure thymic involution had not yet started.”*

f. Please replace "unspecific" with "non-specific" (Materials and methods).

Thank you, this has been corrected.

**Reviewer 2:**

Reviewer #2: Psoriasis and associated autoimmune manifestations are a set of chronic immunopathologies without plenty of available treatment options. Against such a backdrop, the authors have explored a unique side of anti-psoriatic medication, where a certain dose of an RORgammaT inverse agonist ameliorates psoriatic symptoms at the periphery without affecting T cell development. This is definitely of immense clinical importance, since defective thymopoiesis is often associated with several immunosuppressive therapies, such as corticosteroid therapy; unavoidably producing numerous side effects. While I do not find any major drawbacks in the rationale and design of the study, I have a few questions and suggestions towards the authors, as follows.

a. In figure 2, where the authors estimate the effects of their compounds on thymic DP cells in vivo, they have only measured the cell numbers and have shown a steady decrease in it with increasing concentration of their compound. How do they attribute this to be caused by apoptosis and not premature thymic escape of the DP thymocytes (de Meis et al, Journal of Parasitology Research, 2012) without specific assays conducted in vivo which indicate apoptosis (Annexin V binding, TUNEL, cleaved Caspase-3 measurement etc)? The correlation with their in vitro findings does not suffice for such an explanation.

Thank you for this comment. We show in this paper that we induce apoptosis in freshly isolated primary thymocytes ex vivo through AnnexinV staining. While this does not constitute evidence for the same process taking place in vivo, it demonstrates that high levels of inhibition of RORgt leads to apoptosis in the same way as loss of function of RORgt does. That loss of RORgt induces apoptosis in vivo has been established previously, both through AnnexinV staining of thymocytes and TUNEL staining in thymic sections (reference 18, Ueda 2002). Based on these established findings we believe that we can conclude that we lose thymocytes primarily through apoptosis. We have added this reference to row 69 to make this clearer to the reader. Reviewer 1 had a similar comment and hence we have also made changes to the text to reflect this at rows 67-69.

*“In the mouse, thymocytes deficient in RORγt remain hyperproliferative but they also rapidly apoptose through the lack of RORγt-dependent Bcl-xL induced quiescence [16, 18].”*

b. The authors only look at the DP thymocyte number upon treatment with their compounds, and conclude that they are not affecting thymopoiesis to a great extent. However, several recent studies highlight the presence of crucial developmental events which, if perturbed, affect the functionality of the ensuing T cells upon maturation (Gamble et al, Nature Immunology, 2024; Bovolenta et al, PNAS, 2022) without taking a toll on their survival.

Since RORgt has an important role in thymopoiesis, how do the authors ensure that their compounds are not affecting the epigenetic circuitry inside the developing thymocytes in any such way?

This is a good point; however, the paper aims to suggest a potential for a window between the effect on mature effector T-cells and thymus apoptosis. It is likely that with a substantial inhibition of RORγ in the thymus of mice many perturbations to the normal T-cell development can be expected, including epigenetic effects and effects on TCRα splicing and recombination. These can be inferred from the observed sustained proliferation followed by rapid apoptosis vs a proliferative burst followed by quiescence and then more limited apoptosis. Additionally, a skewing of the TCR repertoire has been observed both in mice and man. Some of the underlying causes have been described, like the mentioned lack of Bcl-xL upregulation. If we can circumvent the induced apoptosis, then these other issues can be studied. This has not been accomplished in this work, but we have enhanced the discussion on potential consequences of inhibiting RORγ. There was a similar comment from Reviewer 1 and this was addressed in rows 638-644.

*“An additional complication of inhibition of RORgt has been suggested by Guo et al [51] where they have demonstrated a skewing of the TCRa gene rearrangement and hence limitations to the diversity of the T cell repertoire in mice. While skewing of the T cell repertoire is noteworthy, due to the potentially enhanced risk of developing autoimmune disorders and infection, the T cell leukopenia observed in humans carrying RORC biallelic loss of function mutations, is less pronounced than that observed in mice [19].”*

The methods are described in a slightly haphazard way. While there are plenty of details regarding how the authors validate the screened compounds in terms of their binding capacity, there is insufficient information about which compounds (or library) were deemed qualified for screening, and based on what criteria.

Thank you for this comment. In this paper we have focused on describing biological effects of RORγt inhibition in developing thymocytes and peripheral mature T cells that are of general interest to the field. The chemical development of this series of RORγt inverse agonists have been described in three publications by us (see references 30-32) where a detailed account of the chemical evolution as well as the desired properties are presented. We have updated the introduction rows 100-101 to emphasize that information regarding the chemistry campaign is described in the three papers referenced.

The methodology related to the imiquimod-induced skin inflammation model is also written in a wayward manner. When is the compound applied, before or after imiquimod application?

Thank you for pointing this out. This is an omission on our part and the text has been updated to clarify timing and order of manipulations. Rows 270-271 now reading:

*“The application of Aldara cream took place in the morning after oral compound dosing.”*

The connection between RORgammaT manipulation and thymic lymphoma is very interesting. The authors should mention the risks of thymic T cell progenitor leukemia as well, since dysregulated IL-17 signaling and RORgammaT-dependent pathways are critically involved in the emergence of early T cell progenitor leukemia (Mukherjee et al, Frontiers in Cell & Developmental Biology, 2022).

Thanks for raising these points, it is indeed a very interesting paper and it is supported by other papers. Tarantini et al. Biomarker Research (2021) 9:89 <https://doi.org/10.1186/s40364-021-00347-z>

However, there is no mention of neither IL-17 nor RORγt in either of these papers as being active in ETP as stated by the reviewer. The current data supports that loss of RORγ or RORγt function in the mouse can lead to the development of T-ALL. In reference 14 (Xi Immunity. 2006 Jun;24(6):813-826. doi: 10.1016/j.immuni.2006.03.023), the expression of RORγt has been determined to take place at the DN3 stage and the mechanism whereby first a proliferative burst takes place followed by a quiescent period during which TCRα gene rearrangement takes place has been established. This has been further corroborated and elaborated upon more recently by for example Elife. 2022 Feb 3:11:e75508. doi: 10.7554/eLife.75508. These events all take place in later developmental stages of thymocyte maturation than ETP-ALL, which is considered to originate before the DN3 stage (Tarantini et al. Biomarker Research (2021)). We hope that we have answered this question with the explanation above and given references.

In the discussion, the authors mention the variability in thymus size of 6-8 weeks old mice as a limitation of this study. Again, they mention the age of mice used in the thymic involution experiments as 5 weeks. They need to explain this discrepancy.

Thank you for noticing this. Young mice (before sexual maturation) have not yet begun the natural process of thymic involution when the thymus shrinks in size, weight and cellularity. Since natural thymic involution is variable and indistinguishable from thymic effects of RORγt inhibition we choose to perform our studies in young mice before this process has started. The text has been updated to clarify this at rows 246-248 and 684-685.

The authors test the efficacy of their compounds in an imiquimod-induced dermatitis model, which very weakly resembles psoriasis (Hawkes et al, Journal of investigative dermatology, 2017). This needs to be clearly mentioned in the discussion section.

Thank you for pointing this out to us. The discussion has been updated and additional references have been added as suggested by the reviewer, rows 602-606:

*“It is worth to note that this is one of many animal models used to mimic part of the complex pathophysiology of psoriasis [45]. The model only weakly resembles human psoriasis but allows the evaluation of systemic anti-inflammatory effects targeting RORγt biology [46].”*
