# Supplementary material for: RORγt inverse agonists demonstrating a margin between inhibition of IL-17A and thymocyte apoptosis
Source: PLoS One. 2025 Jan 16;20(1):e0317090. doi: 10.1371/journal.pone.0317090 (PMC11737796; doi:10.1371/journal.pone.0317090)
Supplement: Attachment — Submitted filename: Response to reviewers.docx [file pone.0317090.s008.docx]

ANSWERS TO REVIEWER’S COMMENTS

PONE-D-24-01191R1

**RORγt inverse agonists demonstrating a margin between inhibition of IL-17A and thymocyte apoptosis**

***Editor’s summary and main concerns:***

Thank you for submitting your manuscript to PLOS ONE. After careful consideration, we feel that it has merit but does not fully meet PLOS ONE’s publication criteria as it currently stands. Therefore, we invite you to submit a revised version of the manuscript that addresses the points raised by both the reviewers.

Dear Editor

Thank you for the possibility to revise this manuscript again. We appreciate the reviewers’ time and effort with this, and we can see that the manuscript is now improved and that limitations with the study are now highlighted even more.

We have formulated responses to each of the questions below and updated the manuscript accordingly. We hope that our responses will be satisfactory.

Yours sincerely,

Dr Mia Collins

Review Comments to the Author

**Reviewer 1:**

Reviewer #1: The authors have addressed most of my concerns. I have a minor issue with their response to my comment no. c in the previous round of review. In their response as well as the modification in the manuscript, they do not mention the need for assessing thymocyte apoptosis in the presence of ex vivo stimulation. What they tried to convey through the modified text in the manuscript was not clear to me in this context. Indeed, thymocytes die in the thymus both due to excessively strong TCR stimulation or no stimulation at all. While they have analysed the effects of their compounds on the thymocytes in absence of any stimulation, it would be of greater value if they could assess the same in the presence of ex vivo stimuli. The authors need to give a more to-the-point explanation for this.

Thank you for following up on this comment.

In the normal situation DP thymocytes increase their expression of a fully formed TCRab receptor and begin to sample self peptides presented to them. However, DP thymocytes are dependent on Bcl-xL for their survival, the expression of which is governed by RORgt. Hence, blocking RORgt causes the loss of quiescence and cells begin to apoptose, effectively reducing the number of thymocytes expressing a fully formed TCRab receptor. This is an upstream event before thymocytes commit to becoming CD8+ Tc or CD4+ Th cells and leave the thymus as mature naive T-cells. It is therefore unlikely that exogenous CD3/CD28 stimulation or similar will affect this relatively short assay. In both mouse and human the number of mature peripheral T-cells is reduced in subjects where the RORgt protein function has been lost, but fully mature peripheral T-cells still exist. Apparently, a fraction of T-cells escape the thymus although RORgt has been lost, potentially depending on the timing or the strength of the positive selection signal (i.e. TCR stimulation) and this is an effect that we would suggest warrants further investigation. In this situation the reviewer’s suggestion is important to consider but not entirely straightforward to accomplish in this short assay. The assay is run as separated whole thymus cells, but the critical cellular organization and cytokine milieu is of course severely disrupted and quite difficult to mimic. As a follow up experiment to this assay, we do the in vivo experiment where similar effects are seen in an intact thymus with endogenous physiological TCR stimulation and selection.

Nonetheless, the limitations part of the discussion has been updated to reflect the reviewer’s important point. See rows: 701-706

*“An additional limitation is the artificiality of the thymocyte apoptosis assay where the thymic organization is disrupted thereby limiting the normal cytokine milieu and cellular cross talk (TCR stimulation). To circumvent this the thymocytes could have been stimulated in different ways. However, the enhanced apoptosis is supported in the thymus involution in vivo study where signaling and cross talk are intact.”*

**Reviewer 2:**

Reviewer #2: Collins et al have demonstrated formidable sincerity in addressing my comments. However, some of their responses could not directly address the particular queries raised by me.

1. Apoptosis and thymic escape of DP thymocytes are not mutually exclusive events [de Meis, Juliana et al. “Thymus atrophy and double-positive escape are common features in infectious diseases.” Journal of parasitology research vol. 2012 (2012): 574020. doi:10.1155/2012/574020; Démoulins, Thomas et al. “Reversible blockade of thymic output: an inherent part of TLR ligand-mediated immune response.” Journal of immunology (Baltimore, Md. : 1950) vol. 181,10 (2008): 6757-69. doi:10.4049/jimmunol.181.10.6757]. How do the authors, then, conclude that the loss of DP thymocytes does not involve premature DP escape as a potential reason? Simply referring to the fact that DP apoptosis is predominant during thymic involution does not suffice for this statement.

Thank you for pointing out this important phenomenon. The reviewer correctly points out the lack of evidence to rule out premature thymic escape in our work. Having read the suggested papers and others, the published literature is limited in describing this in non-infectious or non-inflammatory situations, while being clear in establishing that loss of- or inhibition of- RORgt induces a direct and rapid DP thymocyte apoptosis. Given that our experimental animals are healthy and reside in a relatively clean environment, our conclusion is that the majority of the DP loss can be attributed to apoptosis. To highlight that the manuscript does not address this, we have updated the limitations part of the discussion to illuminate this shortcoming, see rows: 695-701

*“A potential complication in measuring thymus cellularity of double positive thymocytes is premature thymic escape. This process has not been investigated in this work, however most of the literature has investigated premature thymic double positive escape in the context of infection or inflammation [61,62]. However, it is established by us and others that loss [16,17] or inhibition of [54] RORγt has a direct and rapid effect on apoptosis. Since these animals are healthy and in a clean environment, we attribute the majority of the rapid loss of double positive thymocytes to apoptosis.”*

2. The authors state that the reference mentioned by me [Mukherjee, Soumyadeep et al. “In Silico Integration of Transcriptome and Interactome Predicts an ETP-ALL-Specific Transcriptional Footprint that Decodes its Developmental Propensity.” Frontiers in cell and developmental biology vol. 10 899752. 13 May. 2022, doi:10.3389/fcell.2022.899752] does not explicitly mention that ETP-ALL has high IL-17 activity. However, figure 2F in that article clearly shows a strong enrichment of IL-17 signaling in ETP-ALL. Furthermore, defined subsets of DN1 thymocytes (which also contribute to ETP-ALL) express a handsome amount of RORC transcript [Spidale, Nicholas A et al. “Interleukin-17-Producing γδ T Cells Originate from SOX13+ Progenitors that Are Independent of γδTCR Signaling.” Immunity vol. 49,5 (2018): 857-872.e5. doi:10.1016/j.immuni.2018.09.010], whereby it cannot be ruled out that RORgammaT inhibition might have some impact on ETP-ALL. I think the authors underestimated the implications of my comment in this context and it would be great if they highlight this aspect with due care.

We clearly underestimated the implications of the reviewer’s comments regarding ETP-ALL and IL-17 and ROR gamma and for this we are sorry. The reviewer is correct in highlighting that ETP-ALL are differentiated from non-ETP-ALL by an IL-17 signal and that DN1 cells can express ROR gamma. We have updated the text to reflect the involvement of this pathway in ETP-ALL, see rows: 590-596

*“However, in a subset of acute lymphoblastic leukemia originating in the early T precursor cell (ETP-ALL), enrichment in the IL-17 pathway has been described [43]. Furthermore, during early thymocyte maturation, in the DN1 stage, a subset of thymocytes upregulate the expression of Rorc [44] and these cells can, if dysregulated, also develop into ETP-ALL. In this situation it may be valuable to evaluate if inhibition of RORyt reduces the proliferation and expansion of such cells.”*

3. 6-8 weeks old mice are barely susceptible to age-induced thymic involution. However, natural age-associated thymic involution starts from approx. 4 weeks. Therefore, the authors’ claim regarding the optimality of their use of 5 weeks old mice for thymic involution experiments does not make much sense. In that case, they need to substantiate their claim by treating 0-2 weeks old pups with IMQ and check its effects on thymic involution. Nevertheless, I believe simply rephrasing their claim might be enough to avoid the confusion.

This is an important point that we apologize for overlooking.

The mouse thymus involution indeed starts at 4 weeks of age. The most rapid decline in the mouse thymus starts after puberty and has a more rapid onset in males than females. In an experimental setting, stress has a far bigger impact on the thymus size at this time why precautions were taken to minimize stressors in the animal facility. We have reworked the text according to the reviewer’s suggestion, please see rows 246-251 which now reads:

*“Female C57BL/6NCrl mice (Charles River, MA, USA) arrived at AstraZeneca at an age of 5 weeks +/- 2 days and were organized into groups and acclimatized for one week in order to reduce stress and variability of thymus weight. The mice in the experiment were intentionally young to reduce the impact of the normal process of thymic involution that starts at 4 weeks as this introduces further variability with regards to thymus size and cellularity. Additionally, age-related thymic involution is at its largest post puberty and less pronounced in females [34].”*

And rows 689-695

*“Furthermore, the already occurring natural thymic involution would introduce further variability in thymus size in the adult animals needed in the IMQ experiment, both for the immune system to be fully matured and to minimize variability from growth related differences in size and thickness of ear pinnas and cell numbers. This limitation did not apply to the experiments studying thymus effects as the animals were intentionally young to minimize effects from early thymic involution.”*
